# Evaluation of a school-based health education program on hepatitis B virus infection prevention practice in rural South-Western, Nigeria

**DOI:** 10.1186/s12889-024-18092-x

**Published:** 2024-02-23

**Authors:** Titilayo Olaoye, Blessing Osie-Efietie, Ololade O. Ogunsanmi, Adebayo M. Mustapha, Ifeoluwapo Asekun-Olarinmoye, Nnodimele Onuigbo Atulomah

**Affiliations:** 1 Department of Public Health, Babcock University, Ilishan Remo-Ogun State, Ilishan, Nigeria; 2 Department of Health and Safety Education, Delta State University, Abraka, Nigeria; 3 University of Medical Sciences, Ondo, Ondo State Nigeria; 4 Department of Health Sciences, Cavendish University, Kampala, Uganda

**Keywords:** Hepatitis B, School-based, Teacher-instructed, Peer-directed, Infection prevention, Southwest Nigeria, Rural communities

## Abstract

**Background:**

Hepatitis B virus (HBV) infection prevention is most effective early in childhood with vaccination programme. However, where this is missed, primary prevention modes of intervention become an alternative recommendation to be considered before the occurrence of risk exposure to the virus. This study sought to evaluate outcomes of a theory-based HBV infection prevention educational intervention among students from four selected secondary schools in Ogun state, Nigeria.

**Methodology:**

A quasi-experimental design enrolling 256 consenting secondary school students from four schools in Ogun state randomized into three intervention schools consisting teacher-instructed (E1), peer-directed (E2) and combination of the two (E3) respectively with a control group ( C) was implemented. The theory-based educational intervention was for six weeks with follow-up period of 8 weeks. A 66-item validated instrument was used to collect data at three reference points and response items for variables in the study were transformed into weighted-aggregate scores of mean and standard deviation of HBV infection prevention practice of participants. Statistical analysis of ANOVA, paired-sample t-test and Cohen’s D Effect Size (ES) was used to quantify the changes produced by the intervention on the outcome variable at 5% level of significance.

**Results:**

At baseline, there was no significant difference (*p* > 0.05) in the mean HBV infection prevention practice scores between the four groups E1 (17.21 ± 3.03), E2 (15.57 ± 1.90), E3 (17.90 ± 3.10), and C (15.20 ± 2.44). However, at 14th week follow up, there was observed significant differences in mean scores of HBV infection prevention practices between all four groups E1 (23.09 ± 2.4), E2 (22.6 ± 3.6), E3 (23.82 ± 2.3), and C (15.25 ± 2.4). Paired-sample t-test conducted demonstrated significant differences between baseline and 14th week follow up for E1 (17.21 ± 3.07 and 23.18 ± 2.9; *p* = 0.001), E2(15.57 ± 1.90 and 23.53 ± 3.12; *p* = 0.001), E3(17.90 ± 3.10 and 25.1 ± 2.6; *p* < 0.001), but not for C (15.20 ± 2.44 and 15.25 ± 2.4; *p* = 0.92), with most significant impact (ES(95%CI) on HBV infection prevention practices observed for E2(3.106 95%CI: [2.66 to 3.55; *p* = 0.001]). Importantly, the participants in E2 showed more improvement in prevention practices than their counterparts from E1, E3, and control. Therefore, the intervention demonstrated proof-of-concept in facilitating behavior modification expected. Peer education can be utilized as a strategy to promote Hepatitis B infection prevention practices among adolescents.

**Supplementary Information:**

The online version contains supplementary material available at 10.1186/s12889-024-18092-x.

## Background

Developing strategies to address the challenges and concerns associated with hepatitis B virus infection among adolescent population in Nigeria who are at high risk of transmission constitute a major public health concern. Adolescents constitute more than 42% of the population in Nigeria and are major contributors to this problem [1, 2]. The second source of concern is that a significant proportion of the country’s adolescents engage in sexual risk behaviors that expose them to the likelihood of an infection and are known to share injection drug-use paraphernalia which promotes HIV infection and hepatitis B virus (HBV) transmission [1, 3] [4].. HBV infection has been a long-standing health risk for advanced liver cancer and outcomes [5].

In a study of HBV infection among sexually active young persons reported a 6.1% prevalence in Abeokuta, Ogun State [3]. In Calabar, an adolescent gender-based study on HBV infection revealed a prevalence of 0.8% for males and 1.8% for females in six secondary schools and a prevalence of 1.0% for children receiving outpatient treatment from a tertiary health center in south-west Nigeria all of a population of young people [6, 7]. Previous studies on hepatitis B infection in the country have primarily focused on disease prevalence [6–9], with few disease-related intervention studies on prevention and control. The few intervention studies conducted in Nigeria have focused on the effect of peer- or instructor-led educational interventions on adolescent knowledge and prevention of hepatitis infection [10–12], paying little attention to their perception or health belief regarding the disease, which is an important pathway to understand the dynamics of the risk behavior involved.

Schools provide excellent opportunities for delivering hepatitis B virus infection educational interventions, and Teachers are known to have a significant impact on students’ lives, not only due to their responsibility to meet their academic needs, but additionally due to their influence in shaping and transforming behaviors in many areas of life [13]. Thus, the school is a place where hepatitis B virus-related factual, clear and age-appropriate information can get to adolescents as a captive audience. The teachers are potent instruments for the dissemination of HBV related information to adolescents and in enhancing their capacities to protect themselves from infection. A peer-led approach benefits young people in particular because they are more inclined to acquire knowledge from and be impacted by their peers. Peer educators who have received the necessary training are frequently viewed as role models who provide security and confidence, allowing health education on sensitive and personal issues to take place. Through active participation, youths can be empowered to take ownership of their own growth and active involvement in their community [14].

Numerous studies have confirmed the efficacy of peer-directed [11–13] and teacher-instructed interventions on Hepatitis B virus infection prevention practices of adolescents [15]. The comparison of the effects of these two interventions on prevention of Hepatitis B virus infection among in-school adolescents is however, yet to be thoroughly investigated, especially structured and guided by behavior theories. Such comparison will help researchers apply the most cost-effective and appropriately designed intervention approach for tackling hepatitis B virus infection. Also, this will enable policymakers to formulate appropriate policy statements concerning the control and prevention of hepatitis B virus infection among adolescents in schools.

**Fig. 1:**
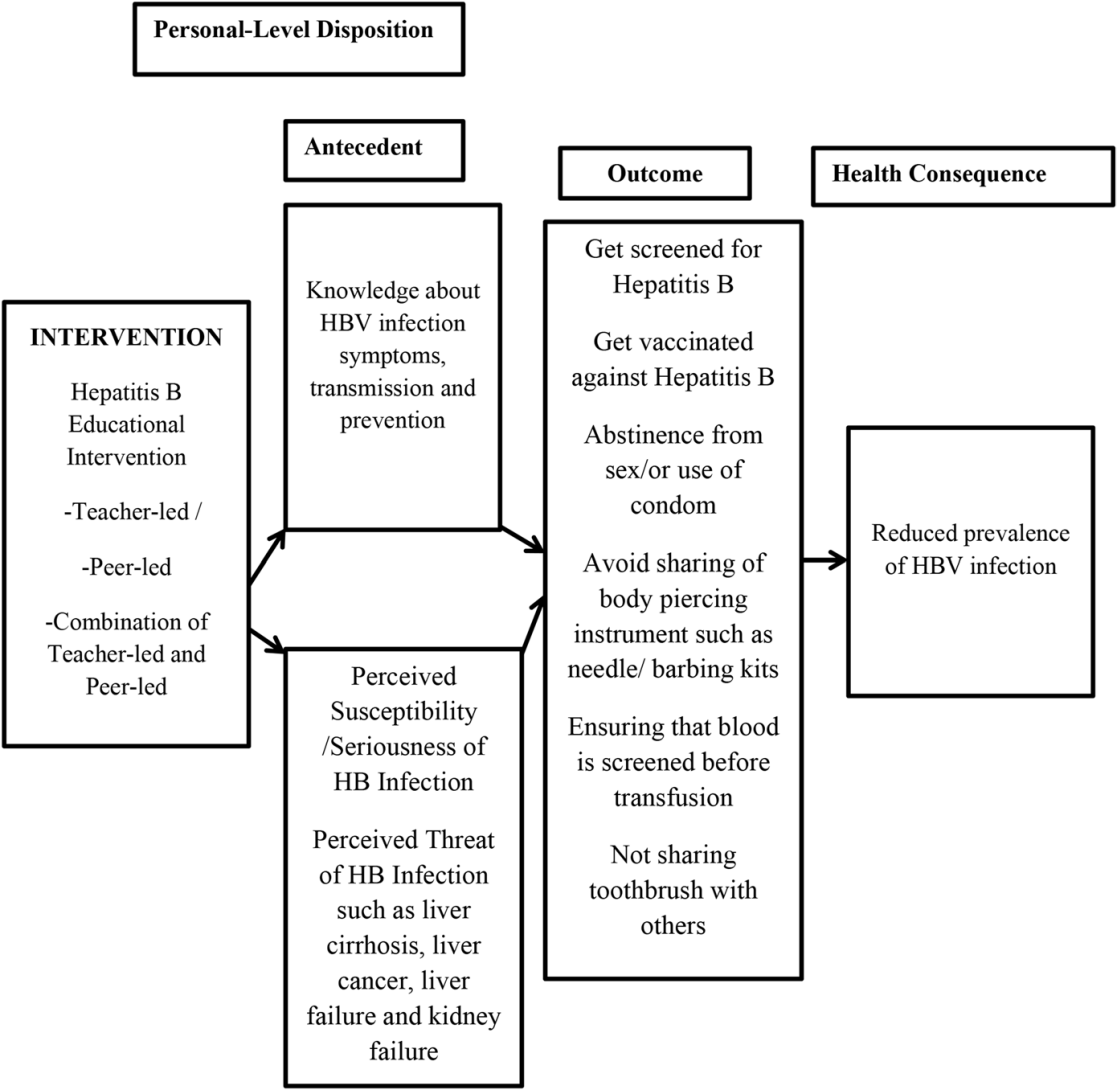
Conceptual framework derived from health belief model guiding the study

Theories and models are very important conceptual resources in health education research and intervention programmes, such as in this study, because they provide the basis for understanding individual behaviors and those antecedent factors that influence risk behavior outcomes, enabling health education programmes to be developed to provide understanding of their dynamics and basis for planning appropriate intervention or proposing likely elucidation of the problem dynamics [16]. Essentially, this study requires theoretical and conceptual clarification for the nature of the behavioral dynamics surrounding achieving HBV infection prevention practices of the at-risk population in this study. The Health Belief Model (HBM), developed by Rosenstock [17] and applied extensively in research, explains preventive health-related behaviors and has been adopted as the theoretical and conceptual framework for this study because of its suitability in elucidating the dynamics in behaviors that carry high risk of adverse health consequences such as HBV infection transmission. Further, the adopted framework has the ability to map out the pathways culminating to health behavior decisions made through computational analysis of an understanding of the cognitive rationale involved and perception of susceptibility and severity of disease as a threat to life, with cost/benefit analysis of treatment as defined by HBM constructs. Thus, HBM explains the theoretical principles underpinning the dynamics of the problem phenomenon and facilitates framing the domain of personal-level dispositions that may be considered the modifiable risk factors the intervention program seeks to address to influence HBV transmission risk behavior (See Fig. 1).

Prevention of HBV transmission and infection of humans can effectively be achieved through primary prevention of health promotion and specific protection offered by vaccination and use of protective devices in procedures that brings persons in direct contact with individuals with the virus in their blood or body fluids. In this study guided by the theoretical framework to establish proof of concept, thus, it is hypothesized that the magnitude of impact of the health education intervention on HBV infection prevention practices of the experimental groups will be significantly better than that of the control group. This study, therefore, sought to investigate the effect of theory-based educational intervention on Hepatitis B virus infection prevention practices involving, teacher-instructed, peer-directed, and a combination of the two programs among in-school adolescents in rural Ogun State, Nigeria.

## Method

### Study setting

Ogun State was created on the 3rd of February 1976, carved out of the old Western State of Nigeria, and named after the *Ogun* River which runs across it from the North to South. Situated between Latitude 6^o^.20’ N and 7^o^ 00’N and Longitude 3^o^ 35’ E, the state covers a total landmass of 16,409.26 sq km. Abeokuta, Ogun state is divided into twenty local government areas and there are five major ethnic expressions among the Yoruba people living in Ogun State. Adolescents and young people make up of 30.7% of Ogun state population [18]. Schools are distributed throughout the state and 90% of the schools are located in rural and sub-urban areas.

### Study design

A pre- and post-test quasi-experimental design was developed that randomized the selected schools into three interventions consisting teacher-instructed (E1), peer-directed (E2) and combination of the two (E3) respectively with one control group (C).

### Study population

Adolescents constituted the population of interest in this study and were selected from government Secondary Schools in Ogun State, Nigeria. There are two thousand eight hundred and fifty-two (20, 850) adolescents in 170 secondary schools in the state comprising two major groups of junior and senior classes.

### Inclusion criteria

Students who consented or provided assent to be involved in the study and ready to complete the study with consent from their parents were enrolled into the programme. Likewise, teachers who gave their consent to participate in the study were also enrolled.

### Sample size and sample technique

The sample size was derived from computations considering 95% confidence level of the standard normal distribution equivalent to Zα = 1.96 accounting for type 1 error and 80% power equivalent to Zβ = 0.84 accounting for type II error, with a maximum tolerable sampling error of 5%. The prevalence rate of HBV infection among Nigeria adolescents aged 11–19 according to Ikobah et al. [6] is estimated at 0.8% for male and 1.8% for female. The computational formula below was applied, thus;

$${\rm{N}}\, = \,{{{{(Z\alpha \, + \,Z\beta )}^2}\, \times \,{\rm{P0}}\,{\rm{(1 - P0)}}} \over {{{\rm{D}}^2}}}$$

Where, N = Sample size, Zα = 95% confidence level of the Standard normal distribution at 1.96, and Zβ = 0.84 at 80% power. P_0_ = Prevalence of hepatitis at 1.8% (Ikobah et al., 2016)). P_1_ = 80% is the desired level of outcome variable in the study, while D = level of precision 0.05. Computing for N;

$${\rm{N}}\, = \,{{{{(1.96 + 0.84)}^2}\, \times \,{\rm{0}}{\rm{.018(1 - 0}}{\rm{.018)}}} \over {{{{\rm{(0}}{\rm{.05)}}}^2}}}$$

$${\rm{N}}\, = \,{{{{(2.8)}^2}\, \times \,{\rm{0}}{\rm{.018(0}}{\rm{.992)}}} \over {{\rm{0}}{\rm{.002}}{{\rm{5}}^2}}}$$

$${\rm{N}}\,{\rm{ = }}\,{{7.84 \times \,0.0176} \over {0.0025}}$$

$${\rm{N}}\,{\rm{ = }}\,{{0.13728} \over {0.0025}}$$

N = 54.9

The minimum sample size is 55.

10% of the minimum sample size was added to take care of attrition.

The total number of participants adding 10% of 55 was = 54 + 5.5 = 60.5 approximately 64.

Hence based on the computation, the minimum total sample size was 256 participants (64 × 4 groups).

Based on sample size calculation, there were 64 participants per experimental group arm.

**Fig. 2:**
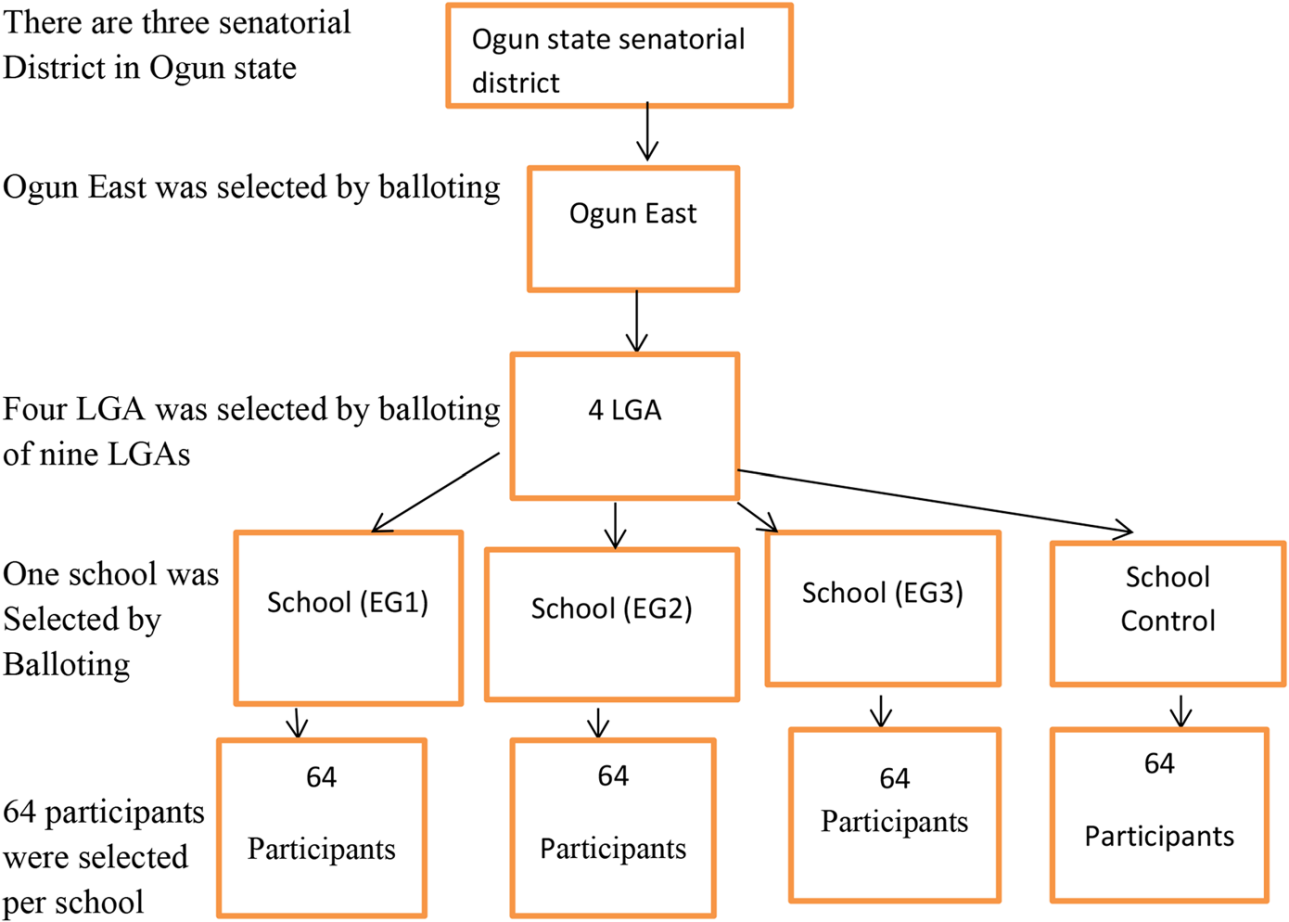
Selection flow chart of multistage outcomes of the participants in the study

Multistage sampling technique was used in the selection of participants who met the inclusion criteria, from Secondary Schools in the Senatorial districts of Ogun State. In the first stage, Ogun East Senatorial districts was selected by simple balloting of one senatorial district out of three in the state. The second stage involved simple random selection by replacement in which four LGA were balloted out of the nine LGAs in Ogun East senatorial district. Similarly, in the third stage, a simple random selection procedure of one government-owned secondary school per LGA selected was conducted through balloting with replacement yielding four schools. The four schools selected were randomized into three intervention groups thus, E1, E2, and E3 with the Control all located within sub-urban and rural settings. In selecting participants in the fourth stage from the four schools, we considered that each school with four levels and each level having five arms with 98 students per arm giving a total of 392 students representing each school, a systematic sampling of the 392 students in the register of the school with sampling interval of 6 provided 64 students from each school. (See, Fig. 2

### Ethical approval and consent to participate

The study protocol was approved by the Babcock University Health Research Ethics Committee and issued an Ethical Research Clearance Certificate with reference number BUHREC397/20. Informed consent was obtained from all subjects and/or their legal guardian(s). Research ethics were strictly observed and maintained throughout the course of the study.

## Measures

A 66-item questionnaire based on the HBM Conceptual Framework guided the construction of the data gathering instrument. The questionnaire collected demographic information, knowledge about hepatitis B virus infectivity, perceptions of hepatitis B virus infection and consequences, and HBV-infection prevention practices. The knowledge items focused on the causative agent for hepatitis, the mode of transmission, symptoms, and methods of prevention of possible infection. Perceived threat of an infection from exposure to hepatitis B virus transmission for the respondents was assessed by a three-response Likert scale of “agreed”, “disagreed”, or had “no opinion” for thirty-five statements regarding hepatitis B virus infection. HBV infection-prevention practices of respondents was contextualized in screening, vaccination against hepatitis B virus, and sexual-risk behaviors related multiple sex partners unprotected and oral sexual intercourse, were assessed by response patterns evaluating frequency (“none at all”, “rarely” “occasionally” and “always”) of performing these behaviors. To establish validity, clarity and understanding of the instrument, a copy of the questionnaire was pre-tested among 20 adolescents from one of the schools in Ogun Southwest senatorial district. Cronbach’s alpha internal consistency of variables in the study ranged between 0.70 and 0.81.

## Procedure for data collection

Data collection was by self-administration procedure in which respondents in the study completed the questionnaires on their own supervised by trained research assistants (RAs) for clarifications and to ensure completion of the items in the instrument. The selected students gathered in designated classrooms in their respective schools where they were instructed on procedures for completing the questionnaires which included preliminary ethical procedures involved in conducting the study. Data was collected at three reference points for all variables in the study at baseline prior to implementing the intervention program, at sixth week immediate-post intervention and 8 weeks after the intervention at fourteenth week follow-up to capture any changes that may have occurred during the follow-up period.

## The intervention

### Design and set-up

The selected schools were assigned into one of the intervention group as follows: teacher-instructed, peer-directed and, combination of peer-directed and teacher-instructed and control. The adoption of the peer-directed and teacher-instructed for this study was motivated by Albert Bandura’s observational learning concept the Social Cognitive theory (SCT). This approach emphasizes that peers through cooperation and information sharing can engage in promoting healthy behavior. Adolescent are more inclined to accept and modify their health-related information and behavior by observing and learning from their mates as compared to adult [19].

Participants were asked to suggest their preferred students and teacher as educators for the hepatitis B virus infection program as part of the intervention planning. Students nominated 8 teachers for the teacher-led group program and 4 for the combination of teacher and peer-led group. These teachers attended three-day training prior to the intervention on communication skills, how the body (liver) works, people at risk of hepatitis B virus exposures, the transmission route, symptoms associated with hepatitis B virus infection, and modalities for HBV infection prevention which was facilitated by the researchers. Methods such as demonstration, role-playing, discussion, and group work were employed during the training sessions. Teachers were assigned to their designated intervention group after completing the training and receiving course materials and teaching materials, as well as forms to document their activities. Each group of eight peer educators received a five-day training program manual with the same content as the teacher but theirs was more intensive than the teachers since they were new in teaching.

### Intervention group

For six weeks, the intervention group received one-hour health education on hepatitis B from peer educators and teacher instructors. The teachers and peer educators utilized discussion and role play method to teach the participants. Also, the session was strictly governed by the researchers’ training manual (curriculum). The training manual (curriculum) was divided into four modules, each with four sessions. The first two modules covered liver function, hepatitis types, hepatitis transmission, clinical features, and recognizing hepatitis B. The third module covered HBV complications and prevention. The fourth module discussed the benefits and, common barriers to preventing HBV. The module was prepared and delivered in English, and it was pre-tested with a sample of 20 students who were not study participants, and was evaluated for necessary corrections and modifications by a health education specialist and a reproductive health specialist.

### Control group

The control group received COVID-19 health education in fulfilment of the ethical requirements, which was designed to last roughly the same period as the hepatitis B virus infection prevention health education. One of the researchers also delivered the modules.

## Statistical analysis

The questionnaires were collected and checked for completeness. The information was coded and entered into a password-protected computer running IBM Statistical Product and Service Solution (IBM SPSS) version 23 to facilitate data analysis. Variables in the study were synthesized from response items by weighted-aggregate scores for knowledge measured on 37-point reference scale, perception on a 35-point reference scale and hepatitis B infection prevention practices measured on 28-point reference scale. Data entered into the computer were reported as summaries of descriptive statistics of means, standard deviation, standard error with test of hypotheses conducted for study impact with paired-sample t-test, ANOVA, Cohen d effect size at 5% level of significance.

## Result

### Demographic characteristics of the participants

Equal number of males and female took part in the study. They were ten to nineteen years old. With 14.56 ± 1.45 years, E3 participants were slightly older than their peers in E1 (14.36 ± 1.68 years), E2 (13.91 ± 1.54 years), and control (14.33 ± 2.00 years).The participants’ were predominantly Christians. Almost all of the participants were from the Yoruba ethnic group. Yoruba represented 84.4% in E1, 89.1% in E2, 81.3% in E3, and 90.6% in control (*p* = 0.12) (See, Table 1).

**Table 1:** Socio-demographic characteristics of participant’s in the control and intervention groups

Variables	Teacher-instructed Education N(%) E1	Peer-directed Education N(%) E2	Teacher-instructed Education & Peer-directed Education N(%) E3	Control N(%)	Statistic	*p*-value
Age(years) $\stackrel{-}{\mathbf{x}}$±SD	**14.36 ± 1.68**	**13.91 ± 1.54**	**14.56 ± 1.45**	**14.33 ± 2.00**	F = 1.71	0.164
**Gender**						
Male	32(50.0)	32(50.0)	32(50.0)	32(50.0)	χ^2^ = 0.000	1.000
Female	32(50.0)	32(50.0)	32(50.0)	32(50.0)		
**Class**						
JSS 3	16(25.0)	16(25.0)	16(25.0)	16(25.0)	χ^2^ = 0.000	1.000
SS1	16(25.0)	16(25.0)	16(25.0)	16(25.0)		
SS2	16(25.0)	16(25.0)	16(25.0)	16(25.0)		
SS3	16(25.0)	16(25.0)	16(25.0)	16(25.0)		
**Religion**						
Christianity	52(81.2)	57(89.1)	46(71.9)	47(73.4)	χ^2^ = 11.37	0.07
Islam	12(18.8)	6(9.3)	18(28.1)	17(26.6)		
Traditional	0(0)	1(1.6)	0(0)	0(0)		
**Ethnicity**						
Yoruba	54(84.4)	57(89.1)	52(81.3)	58(90.6)	χ^2^ = 14.05	0.12
Igbo	8(12.4)	4(6.3)	4(6.2)	5(7.8)		
Hausa	1(1.6)	1(1.6)	1(1.6)	1(1.6)		
*Others	1(1.6)	2(3.0)	7(10.9)	0(0)		

Knowledge, Perception and Prevention practice of Hepatitis B Virus Infection

### Personal-level dispositions of the respondent at baseline

The study measured personal-level disposition of participants to include knowledge, perceptions, and HBV infection prevention practices. At baseline, the results showed that level of knowledge regarding HBV infection issues measured on a 37-point scale for knowledge reported mean scores for participants in E1, E2, E3 and C were 15.58 ± 5.0, 16.75 ± 4.6, 16.20 ± 4.7 and 16.34 ± 3.1 respectively demonstrating no significant difference (*p* = 0.520) across the groups. Similarly, the level of perception measured on a 35-point scale reported no significant difference for participants across the groups (*p* = 0.210). However, the level of HBV infection prevention practices measured on 28-point scale showed a significant difference in mean scores was observed across the groups (*p* = 0.001) with participants in E3 reporting the highest mean score of 17.90 ± 3.10 (See Table 2).

**Table 2:** Mean scores for Hepatitis B virus related knowledge, perception and infection prevention practices in the control and intervention groups at baseline

Variables	Teacher-Led Education E1	Peer-Led Education E2	Teacher-Led Education & Peer-led Education E3	Control	F-Statistic	*p*-value
Knowledge	15.58 ± 5.0	16.75 ± 4.60	16.20 ± 4.70	16.34 ± 3.10	0.760	0.520
Perception	17.78 ± 4.14	16.23 ± 5.35	17.53 ± 3.60	17.05 ± 3.76	1.501	0.210
Preventive practice	17.21 ± 3.07	15.57 ± 1.90	17.90 ± 3.10	15.20 ± 2.44	14.95	0.001

### Personal-level dispositions of the respondent at 14th week follow-up

The results from pairwise comparison at 14th week follow-up of mean scores for personal-level dispositions of the participants for all groups except Control regarding HBV infection and its prevention between baseline and 14th week Follow-up demonstrated significant differences. (See Table 3).

**Table 3:** Comparison of mean scores for Hepatitis B related knowledge, perception and prevention practices baseline and after the intervention

Variables	Teacher-instructed education* $\stackrel{-}{\mathbf{x}}$ ±SD		*p*-value	Peer-directed education* $\stackrel{-}{\mathbf{x}}$ ±SD		*p*-value	Teacher-instructed and peer-directed education* $\stackrel{-}{\mathbf{x}}$ ±SD		*p*-value	Control* $\stackrel{-}{\mathbf{x}}$ ±SD		*p*-value
Time	*Before*	*After*		*Before*	*After*		*Before*	*After*		*Before*	*After*	
Knowledge of Hepatitis B	15.58 ± 5.0	33.90 ± 2.4	0.001	16.75 ± 4.6	33.40 ± 2.1	0.001	16.20 ± 4.7	34.92 ± 1.7	0.001	16.34 ± 3.1	16.40 ± 3.1	0.32
Perception of threat of hepatitis B	17.78 ± 4.14	30.59 ± 2.3	0.001	16.23 ± 5.35	30.31 ± 3.0	0.001	17.53 ± 3.60	31.45 ± 4.5	0.001	17.05 ± 3.76	17.09 ± 3.8	0.32
Prevention practices of hepatitis B	17.21 ± 3.07	23.09 ± 2.4	0.001	15.57 ± 1.90	22.6 ± 3.6	0.001	17.90 ± 3.10	23.82 ± 2.3	0.001	15.20 ± 2.44	15.25 ± 2.4	0.92

*Sample size for all groups (*n* = 64); Paired-T-test was used for baseline and after intervention analysis of difference

### Impact of the intervention on the outcome variable at 14th week follow-up

The effect size (ES) with their respective 95% confidence intervals computed for HBV infection prevention practices in the experimental groups E1, E2, E2 are 2.02(1.50 to 2.53), 3.11(2.66 to 3.55), and 2.54(2.05 to 3.03) whereas, ES for control is 0.02(-0.395 to 0.437). This results demonstrates clearly that the intervention directed by peer educators reported the strongest influence on HBV infection prevention practices of participants in the study with the least impact in the control group (See, Table 4).

**Table 4:** Intervention effect on participant’s Hepatitis B virus infection knowledge, perception, and prevention practices

Variable	Time	Teacher-led education (*n* = 64) $\stackrel{-}{\mathbf{x}}$ ±SD	Peer-led education (*n* = 64) $\stackrel{-}{\mathbf{x}}$±SD	Teacher-led and peer-led education (*n* = 64) $\stackrel{-}{\mathbf{x}}$ ±SD	Control (*n* = 64) $\stackrel{-}{\mathbf{x}}$ ±SD
Knowledge of hepatitis B	Baseline	15.58 ± 5.0	16.75 ± 4.6	16.20 ± 4.7	16.34 ± 3.1
	Follow up	34.67 ± 1.9	34.10 ± 2.1	35.79 ± 1.3	16.45 ± 2.9
	Paired sample T-test results	*P* = 0.001	*P* = 0.001	*P* = 0.001	*P* = 0.44
	*ES (95%CI)	5.50(-5.71to 4.40)	4.85 (-5.47 to 4.24)	5.65(-6.24 to 5.04)	0.03(-0.55 to -0.48)
Perception of threat of hepatitis B	Baseline	17.78 ± 4.14	16.23 ± 5.35	17.53 ± 3.60	17.05 ± 3.76
	Follow up	33.40 ± 1.3	32.46 ± 1.9	33.48 ± 1.6	17.09 ± 3.7
	Paired sample T-test results	*P* = 0.001	*P* = 0.001	*P* = 0.001	*P* = 0.32
	*ES (95%CI)	5.11(-5.64to 4.58)	4.06(-4.75 to3.37)	5.75(-6.23 to 5.27)	0.01(-0.66 to -0.64)
Prevention practices of hepatitis B	Baseline	17.21 ± 3.07	15.57 ± 1.90	17.90 ± 3.10	15.20 ± 2.44
	Follow-up	23.18 ± 2.9	23.53 ± 3.12	25.1 ± 2.6	15.25 ± 2.4
	Paired sample T-test results	*P* = 0.001	*P* = 0.001	*P* = 0.001	*P* = 0.92
	*ES(95%CI)	2.02(1.50 to2.53 )	3.11(2.66 to 3.55)	2.54(2.5 to 3.03)	0.021(-0.40 to 0.44)

*ES: Effect size computed from Cohen’s d

## Dscussion

This study investigated the effect of school-based health education Program on hepatitis B infection prevention practices of in-school adolescents in Rural South-Western, Nigeria. The intervention modality adopted for this study was the educational and behavioral intervention structured around school-based Health Education Program and was factorial in design. This study sought to investigate the effect of theory-based educational intervention on Hepatitis B virus infection prevention practices involving, teacher-instructed, peer-directed and, a combination of the two programs as factorials considered among in-school adolescents in rural Ogun State, Nigeria. The study hypothesized that the magnitude of impact of the health education intervention on HBV infection prevention practices in the experimental groups will be significantly better than that observed in the control group.

The findings from this study has demonstrated that the intervention applied had significant influence on the moderating variables of knowledge and perceptions regarding HBV infection dynamics of adolescents in the study as there was notable changes between pre-intervention and 14th week follow-up understanding of the infection dynamics and its consequences expressed in their knowledge and perception mean scores. The effectiveness of the intervention can be attributed to the design adopted guided by the Health Belief Model that established the proof of concept sought. The theoretical pathways through which infection prevention practices was achieved is explainable by the theoretical framework. The model posits that behaviour exhibited in this study, HBV infection prevention practices, is determined by whether the individual perceives the likelihood of an exposure to the virus with a serious health consequences that threatens quality of life, and that the individual is convinced that treatment or prevention activities are effective and at the same time inexpensive, and receives some form of cue to take health promoting action, would trigger decisions to take recommended health promoting actions aroused by comprehension of the dynamics involved. It should be borne in mind that acceptance of personal susceptibility to a condition varies from person to person, so also is the individual’s perception of the seriousness of the outcomes of exposure. Perception must be viewed in the context of the knowledge of the condition that confronts the individual and how significantly his health literacy, culturally set values and beliefs impinge on this knowledge. These modifying factors may awaken or subdue threats of the likelihood of serious consequences as a result of inactivity. However, the structured health education program considered these in the conceptualization to validate the assumptions that adolescents may be more responsive to cognitive instructions coming from their peers than others.

The participants investigated in this study are secondary school students and the intervention implemented is conceived as modifying factorial designs of teacher-instructed, peer-directed and the combination of both teacher-peer led intervention implemented to answer the question of which would produce the most impact on their HBV infection prevention practices and validate the claim that peer-education almost always produces the best results. In this study notably, peer-directed intervention had a profound impact on HBV infection prevention practices of participants in E2 with an Effect Size of 3.11 95% CI:[2.66–3.55;*p* < 0.05]. This observed changes produced can be interpreted as the peer-directed intervention having a 1.54 magnitude of improvement over E1, 1.22 magnitude of improvement over E3 and 155 magnitude differences over the control as ratios of their ES compared with E2. One would have thought that the effects of the combined E1 and E2 as in intervention E3 would have produced an additive interactions or synergistic result and provide better result, but this did not happen.

Findings in this study is consistent with the finding of Amaugo [20], Borawisk et al., [21] and Rohrbach et al. [22] who observed that knowledge can be increased after educational intervention. The impact of peer-education interventions, by increasing knowledge of adolescents, have previously been demonstrated in studies by Matthew et al. [11], Yakubu et al. [12], and Baghianimogbadam et al. [15]. At baseline of our study, more than half of the adolescents had a fair cognitive understanding of HBV. The results from the intervention demonstrated noticeable differences in the teacher-instructed and peer-directed groups than in the others which were maintained till the end of the 8th -week follow-up period. Gaining knowledge is not only encouraging, but also necessary, because gaining knowledge is typically the first step along the pathway of changing the behavior of an individual. The effect of teacher-instructed and peer-directed interventions and specifically, Hepatitis B virus infection prevention education on the level of knowledge of adolescents have been established in previous studies [10–12, 21]. However, the intervention programme that combined teacher-instructed and peer-directed interventions had a greater impact on adolescents’ knowledge of HBV in these studies. This study suggested that efforts to improve Hepatitis B knowledge through tailored school-based educational programs can have a positive impact on in-school adolescents’ Hepatitis B knowledge. These findings provide validity of principles for the Health Belief Model, which includes knowledge as one of the modifiable contributing factors in responding to a health problem, influencing behavior and stimulating the impetus to act [19].

Similarly, teacher-instructed, and peer-directed intervention had a significant impact on the students’ perception of Hepatitis B infection. This supports previous research findings that teacher- instructed and peer-directed interventions can improve secondary school students’ perceptions of Hepatitis B [11, 12, 22, 23].. Related finding was reported among secondary school students in Iran that teacher-instructed and peer-directed education positively influenced the perception of students towards HIV/AIDS prevention [15]. This change in perception was encouraging since the key factors that influence health behaviours according to the Health Belief Model are the individual’s perception of severity of the disease, potential benefits, and barrier to the behaviour [24]. As a result, school-based programs aimed at changing perceptions would facilitate adolescents recognize their vulnerability and the potential danger of the disease, thereby increasing their likelihood to act on what they have studied. The interventions had a positive impact on the preventive practices of the adolescents. However, the peer-led intervention had higher effect size on the adolescents Hepatitis B related prevention practice compared with the teacher-led and the combined interventions. One explanation in support of the observed dynamics elucidating the success recorded for peer-led intervention is that adolescents in this study are more likely to exhibit more confidence with their peers than would be to discuss preventive practices with their teachers. This supports the findings of studies in Jos [11], Northern Nigeria [12] and in Saudi Arabia [25] that peer education is effective in improving Hepatitis B related practices. Similarly, findings from the work of Baghianimogbadam et al. [15]., corroborates the results in this study. Acemoglu et al. [25] reported that peer education programmes are expected to have a strong influence on the behaviour of the individual adolescent. This implies that to influence positive change in adolescents’ preventive practices, school curriculum must be integrated with interactive approaches such as peer education. However, Liu et al. [26]., found that the teacher-led group improved more than the peer-led group in terms of outcomes of TB prevention practice in China. An explanation for the observed disparity in findings could be attributed to cultural differences.

## Conclusion

This study provides the evidence sought for to suggest that peer-delivered health educational intervention programs have the potential to substantially improve Hepatitis B virus infection prevention practices, or any other risk-related behaviors, of in-school adolescents as clearly demonstrated with the data reported for effect size computed. No doubt, the findings of this study demonstrate that, with adequate training and supportive supervision, teachers and students can be effectively used as change agents in the school environment to implement high-quality programs. Most secondary schools in Nigeria have the human resources required to implement hepatitis B virus infection prevention programs and replicate this throughout the country. Hypothetically, the intervention is likely to have the greatest chance of success in a school setting in which teachers and students play complementary roles; this concept supports the notion that multiple interventions used in tandem can improve the effectiveness of a program. The importance of peer education in hepatitis B virus infection prevention should not be overlooked as the peer-directed had the greatest effect on the adolescent’s prevention practices as compared with other intervention.

### Limitation

There are observed weaknesses in the study and are outlined below:

1. The responses on prevention practices were based solely on self-reported practices.
2. The findings of the results may be peculiar to a particular group of adolescents who may have different practices compared to some other areas in which adolescents are culturally or religiously marginalized. Hence the ability to generalize these findings to other adolescent populations may be limited.

### Implication of the study to public health research and practice

The study shows that although any intervention is better than nothing, some are greater than others. The combination of teacher-led and peer-led intervention protocol has the potential of producing synergy, if designed carefully to operate as additive fractions in which teachers and students peer educators reinforced the efforts of each other. If the adolescent missed HBV vaccine as an infant there is an opportunity to learn about the prevention practices in the school utilizing peer-educators. This tool is affordable and can help reach a large audience.

### Electronic supplementary material

Below is the link to the electronic supplementary material.

Supplementary Material 1

## Data Availability

The data collected and analyzed to generate the results in this study are all within the paper and supporting information files.
