# Supplementary material for: Evaluation of a school-based health education program on hepatitis B virus infection prevention practice in rural South-Western, Nigeria
Source: BMC Public Health. 2024 Feb 23;24:591. doi: 10.1186/s12889-024-18092-x (PMC10893707; doi:10.1186/s12889-024-18092-x)
Supplement: Supplementary file 1 — Supplementary Material 1 [file 12889_2024_18092_MOESM1_ESM.pdf]

Questionnaire

Dear Respondents

The purpose of this study is to collect essential information on knowledge, Perception and Prevention Practices of Hepatitis B among In-school adolescents in Ogun State. The information gathered is going to be used to design an intervention that aims to reduce hepatitis B infection among In-school adolescents. Participation is voluntary and honest answers are desired. All information will be treated with a high level of confidentiality. Do not write your name on any part of the questionnaire. Data collected would solely be used for academic purpose and are assured of total confidentiality of provided information. Your willingness to answer these questions implies you have consented to participate in this study

Thank you for your cooperation.

Kindly indicate a willingness to participate by ticking [√] in the BOX ☐.

Section A: Demographic Characteristics

Kindly complete the blank spaces provided or tick [√] the appropriate responses/answers which concern you in the boxes provided below

1. Name of School____
2. Age as at last birthday _________________ years
3. Gender: 1. Female ☐ 2. Male ☐
4. Class: 1. JSS 3 ☐ 2. SS 1 ☐ 3. SS 2 ☐ 4. SS 3 ☐
5. Religion: 1. Christianity ☐ 2. Islam ☐ 3. Traditional ☐ 4. Others (please specify)________________
6. Ethnicity: 1.Yoruba ☐ 2. Igbo ☐ 3. Hausa ☐ 4.Others(please specify)_______________

Section B: Knowledge of Hepatitis B Infection

Kindly tick [√] the appropriate responses/answers in the boxes provided; you can tick [√] more than one options in some cases.

7. Which one of the following micro-organism causes Hepatitis B infection?

1. Protozoa ☐ 2. Bacteria ☐ 3. Fungi ☐ 4. Virus ☐ 5. I don’t Know ☐

8. Which organ or part of the body is usually affected by hepatitis B virus.

1. Kidney ☐ 2. Liver ☐ 3. Lungs ☐ 4. Brain ☐ 5. I don’t know ☐

Instruction: Table 1 contains a list of risk factors or harmful practices that can aid the spread of hepatitis B infection. For each, please tick [√] whether it is False, True or I don't know.

9	**Practices**	**True**	**False**	**I Don't Know**
9.1	Eating certain foods like garlic and ginger			
9.2	Sweat			
9.3	Sharing blade			
9.4	Drawing of body Tattoos			
9.5	Drinking alcohol			
9.6	Unprotected sexual intercourse			
9.7	Sharing needles			
9.8	Shaking hands with an infected person			
9.9	Infected mother to infant during pregnancy			
9.10	Sleeping on infected person's bed			
9.11	Unscreened blood transfusion			
9.12	Taking Blood oath			
9.13	Sharing of toothbrush			

Instruction: Table 2 contains a list of preventive measures or practices that can help to prevent hepatitis B infection. For each, please tick [√] whether False, True or I don't know.

10	**Hepatitis B infection can be prevented by;**	**True**	**False**	**I don't Know**
10.1	Taking vaccine against hepatitis B			
10.2	Engaging in regular exercise			
10.3	Eating appropriate/balance diet			
10.4	Washing of hands always			
10.5	Using condom during sexual intercourse			
10.6	Not sharing injection needles with others			
10.7	Sharing blade with friends and family members			
10.8	Using one's personal barbing kits			

11. Which one of the following can be used to find out whether someone has hepatitis B or not? (Tick [√] the correct answer)

1. Facial appearance☐ 2. Blood test ☐ 3.Urine test ☐ 4. Sputum test ☐ 5. I don't know ☐

12. Which of the following can hepatitis B lead to? (Tick [√] all the ones you consider to be correct).

1. Coronavirus disease ☐ 2. Liver cirrhosis ☐ 3.Malaria ☐ 4. Cancer of the Liver ☐ 5. I don't know ☐

13. A person can have hepatitis B without showing symptoms of the disease. 1. True ☐ 2. False ☐ 3. I don't know ☐

14. What are the **possible symptoms** of hepatitis B infection?(Tick [√] all ones you consider to be correct).

1. Abdominal pain☐ 2. Jaundice ☐ 3. Tiredness ☐ 4. Headache ☐ 5. I don't know ☐

15. If someone has hepatitis B virus, the virus remains in the person body for life. 1. True ☐ 2. False ☐ 3. I don't know ☐

16. How many hepatitis B vaccine injections should one take in order to be fully protected against the virus?

1. Four ☐ 2. Two ☐ 3. Three ☐ 4. One ☐ 5. I don't know ☐

17. Which of the following groups of people should be given the hepatitis B vaccine (tick [√] all that you consider to be correct).

1. Babies ☐ 2.Adolesscents who are yet to receive the vaccination ☐ 3. Health workers ☐ 4. People who share drug injection equipment ☐ 5. Anyone who wants to be protected against hepatitis B infection ☐ 6. I don't know ☐

Section C: Perception of Adolescents on Hepatitis B Infection

Table 3 shows the different perception which some people have concerning hepatitis B infection. For each perception tick [√] whether you Agree (A)or Disagree (D) with it; if you have not made up your mind tick No Opinion Yet (NOY).

S/N	Perception of hepatitis B infection	A	D	NOY
18	**Perceived Susceptibility**			
18.1	Not everyone can get hepatitis B			
18.2	Hepatitis B cannot infect people who have faith in God			
18.3	Vaccination against Hepatitis B is not necessary for me because I'm a child of God			
18.4	I am sure I have full protection against hepatitis B infection			
18.5	I am very careful when using sharp objects(especially needle or blade) that I borrow from friends			
18.6	Hepatitis B cannot be transmitted from an infected mother to her child during childbirth.			
18.7	It is people who have sex with more than one boy/girlfriends that can get hepatitis B			
18.8	Having tattoo on the body can put you at risk of contracting hepatitis B infection			
18.9	Consumption of alcoholic beverages (beer, gin and wine) can make one get hepatitis B infection			
18.10	Taking one's clipper/needles to the salon can reduces one's risk of getting hepatitis B			
18.11	It is people who share barbing clipper with others in the barbing salon that can get hepatitis B			
18.12	Touching the body fluids (blood) of person who have hepatitis B infection cannot make one to have hepatitis B if one has strong immunity			
19	**Perceived Seriousness/Severity**			
19.1	Hepatitis B is a very serious disease			
19.2	Hepatitis B can lasts for a lifetime in someone's body			
19.3	Hepatitis B cannot damage the liver			
19.4	One can always recover from the disease			
19.5	Hepatitis B cannot lead to death			
19.6	Hepatitis B can be cured.			
19.7	Eating a healthy diet can prevent hepatitis B			

19.8	Hepatitis B is a mild disease			
19.9	Regular exercise cannot prevent hepatitis B			
20	**Perceived Benefit of preventing hepatitis B infection**			
20.1	Screening for hepatitis B makes one knows whether he/she is infected with hepatitis B or not.			
20.2	Hepatitis B infection is preventable			
20.3	Taking hepatitis B vaccine early enough to protect oneself can save one from spending a lot of money in hospital for treating hepatitis B infection			
20.4	Taking hepatitis B vaccine can protect people from spreading hepatitis B infection in the community			
20.5	Using a condom during sex cannot prevent one from having hepatitis B infection.			
20.6	A pregnant woman who has previously vaccinated against hepatitis B do not have the risk of transmitting hepatitis B infection to her foetus or child			
20.7	Abstaining from sex can protect one from hepatitis B infection			
21	**Perceived Barrier**			
21.1	Lack of knowledge about hepatitis B infection can make people to refuse to go for hepatitis B screening.			
21.2	The screening is too expensive.			
21.3	Location of hospital can prevent someone from going for hepatitis B virus screening.			
21.4	I think the vaccine cost is too much			
21.5	Fear of the side effects of the vaccine is good reason for not taking hepatitis B vaccine.			
21.6	Not having time can prevent someone from going for screening			
21.7	Fear of needle can prevent someone from getting vaccinated			

Section D: Adolescents Prevention Practices Concerning Hepatitis B

22. Have you been screened for hepatitis B before? 1. Yes ☐ 2.No ☐ 3.Cannot remember ☐

23. Have you ever received a hepatitis B vaccine before? 1. Yes ☐ 2. No ☐ 3. Cannot remember ☐

24. How many doses of hepatitis B vaccine did you receive? 1. None ☐ 2. One ☐ 3. Two ☐ 4. Three ☐ 5. Other (please specify)____________________

25. Would you be willing to be tested for hepatitis B infection? 1. Yes ☐ 2. No ☐

26. Have you ever had sex? 1. Yes ☐ 2. No ☐

27. How old were you when you first had sex? _________________________

28. Do you have sex in the last six month? 1. Yes ☐ 2. No ☐ 3. Never had sex ☐

29. How many girl/boyfriend do you have? 1. One ☐ 2. Two ☐ 3. Three ☐ 4. Four and above ☐ 5 None ☐

30. How many girl/boyfriend do you currently have sexual intercourse with? 1. One ☐ 2. Two ☐ 3. Three ☐ 4 Four and above ☐ 5. Never had sex ☐

31. Do you use a condom during your last sexual intercourse? 1. Yes ☐ 2. No ☐ 3. Never had sex ☐

32. Have you ever used condom? 1. Yes ☐ 2. No ☐

33. If you use condom, what disease or thing do you use condom to prevent? (Tick [√] all the things or diseases you use condom to prevent). 1. HIV/AIDs ☐ 2. Sphills ☐ 3. Gonorrhoea ☐ 5. Hepatitis B ☐ 6. Chlamydia ☐ 7. Pregnancy ☐ 8. Never had used condom ☐

34. In the last 6 weeks how often do you use condoms? 1. Very often ☐ 2. Often ☐ 3. Occasionally ☐ 4. Rarely ☐ 5. Never had sex ☐

35. Do you have a barbing kit of your own? 1. Yes ☐ 2. No ☐

36. Do you share blade, injection, toothbrush or any body piercing tools with your family members? 1. Yes ☐ 2. No ☐

37. In the last 6 weeks how often do you share blade, injection, toothbrush or any body piercing tools with your family members? 1. Very often ☐ 2. Often ☐ 3. Occasionally ☐ 4. Rarely ☐ 5. Never ☐

38. Do you share blade, injection, toothbrush or any body piercing tools with friends? 1. Yes ☐ 2. No ☐

39. In the last 6 weeks often do you share blade, injection, toothbrush or any body piercing tools with your friends? 1. Very often ☐ 2. Often ☐ 3. Occasionally ☐ 4. Rarely ☐ 5. Never ☐

40. Do you have a tattoo on your body? 1. Yes ☐ 2. No ☐

41. Are you planning to have a tattoo on your body? 1. Yes ☐ 2. No ☐

Thank you for your participation
